# Early Growth Parameters as Predictors of Developmental Delay among Children Conceived During the 2015–2016 Zika Virus Outbreak in Northeastern Brazil

**DOI:** 10.3390/tropicalmed5040155

**Published:** 2020-10-01

**Authors:** Charles E. Rose, Jeanne Bertolli, Jacob Elijah Attell, Cynthia A. Moore, Flavio Melo, Kim Kotzky, Nevin Krishna, Ashley Satterfield-Nash, Isabela Ornelas Pereira, Andre Pessoa, Donna Camille Smith, Ana Carolina Faria e Silva Santelli, Georgina Peacock

**Affiliations:** 1 National Center on Birth Defects and Developmental Disabilities, Centers for Disease Control and Prevention (CDC), Atlanta, GA 30329-4027, USA; cvr7@cdc.gov (C.E.R.); cam0@cdc.gov (C.A.M.); 2 Division of Human Development and Disability, CDC, Atlanta, GA 30329-4027, USA; ghn3@cdc.gov; 3 Eagle Global Scientific, SanAntonio, TX 78248-4534, USA; attell_jacob@bah.com; 4 Hospital Regional de Guarabira/Governo do Estado da Paraíba, João Pessoa, Paraíba 58225-000, Brazil; flaviopediatra@yahoo.com.br; 5 Oak Ridge Institute for Science and Education, Oak Ridge, TN 37830-8007, USA; kkotzky@gmail.com (K.K.); dr.ashley.nash@gmail.com (A.S.-N.); 6 Center for Preparedness and Response, CDC, Atlanta, GA 30329-4027, USA; hbx0@cdc.gov; 7 Department of Chronic Conditions and Sexually Transmitted Infections (DCCI), Ministry of Health of Brazil, Brasilia 70719-040, Brazil; bela.op@gmail.com; 8 Pediatrics Department of Ceara State Universitiy—UECE, Fortaleza, Ceará 60714-903, Brazil; andrepessoa10@yahoo.com.br; 9 Division of Congenital and Developmental Disorders, CDC, Atlanta, GA 30329-4027, USA; dcamillesmith@protonmail.com; 10 Division of Global HIV and TB, Center for Global Health, CDC, Atlanta, GA 30329-4027, USA; nuw6@cdc.gov

**Keywords:** congenital Zika virus infection, growth and development, developmental disabilities

## Abstract

Background: Identifying infants with congenital infection for early intervention will likely be challenging in future Zika virus outbreaks. We investigated indicators of risk for developmental delay among children born with and without obvious manifestations of congenital Zika virus infection. Methods: We evaluated 120 children conceived during the 2015−2016 Zika virus outbreak in Paraíba, Brazil. We analyzed data from children at birth; ages 1−7 months and approximately 24 months, using medical records (i.e., anthropometric measurements diagnoses), medical evaluation (i.e., Zika/other laboratory tests, dysmorphic features), and parent report (seizures, developmental delay). We used a Bayesian modeling approach to identify predictors of developmental delay. Results: Head circumference (HC) and length at birth and rates of growth for HC and length at follow-up were consistent across domains of developmental delay; (e.g., for every 1 cm per month decrease in HC growth rate; there was a corresponding decrease in the gross motor *z*-score). Modeling results indicated that HC and length at birth, and follow-up HC and length rates of growth, were predictive of developmental delay. Conclusion: These findings suggest that accurate measurement and frequent monitoring of HC and length, especially in the first few months of life, may be useful for identifying children possibly congenitally exposed to Zika virus who could benefit from early intervention services.

## 1. Introduction

Zika virus infection has waned since the 2015 outbreak began in South America, but has the potential to continue in a cyclical pattern, such as dengue, following the periodicity of population immunity [1,2]. The performance of diagnostic tests presented challenges during the recent Zika outbreak, for identifying both individual children with congenital Zika infection and the range of other infection-related outcomes in children [3]. Microcephaly and severe brain abnormalities were the initially recognized indicators of congenital Zika virus infection [4,5,6,7,8,9,10,11,12]. Subsequent reports described children with confirmed intrauterine Zika virus exposure who were born without microcephaly or other obvious brain pathology, and either have postnatal-onset microcephaly, or were determined to have neurodevelopmental abnormalities without microcephaly [10,11,12,13,14,15,16,17,18,19]. Whether the observed less severe neurodevelopmental abnormalities in congenitally exposed children without microcephaly can be attributed to Zika virus infection is an open question. Regardless of etiology, such children may not be identified early enough to benefit maximally from interventions without appropriate developmental monitoring. A head ultrasound by one month of age is currently recommended for infants without clinical findings consistent with congenital Zika syndrome who were congenitally exposed to Zika virus [17], but ultrasound is not designed for identifying functional deficits. Early indicators of developmental delay are necessary to identify children who may benefit from timely intervention services in future outbreaks.

Investigators have noted that congenital Zika infection affects infant growth, especially head circumference (HC), and have observed variability in degree and timing of HC changes as an indicator that an infant has been infected [9,10,11,12,13]. Some research suggests that congenital Zika infection may affect body length [12,13], although Walker et al. [20] describe a femur-sparing pattern of abnormal fetal growth associated with maternal Zika virus infection. There is evidence that congenital infections with other neurotropic viruses affect growth (e.g., HC, length, weight), and that these early growth parameters are predictive of cognitive impairment later in childhood [21,22] However, to inform monitoring and care of infants born during and following future Zika virus outbreaks, research could help to determine if early growth patterns might be useful as predictors of later functional deficits.

To address this research gap, we investigated whether early growth parameters predict developmental delay following a Zika outbreak using data from a follow-up investigation of children conceived in northeastern Brazil during the 2015−2016 Zika virus outbreak [23]. These children were selected from population-based registries for participation in a 2016 case-control study to investigate the association between microcephaly and congenital Zika infection. We identified growth parameters, measurable in the first six months of life, that are predictive of developmental delay manifested by age two years. 

## 2. Materials and Methods 

### 2.1. Study Population

The Zika Outcomes and Development in Infants and Children (ZODIAC) investigation was a follow-up evaluation of children conceived during the 2015−2016 Zika virus outbreak in northeastern Brazil, who had participated in a 2016 case-control study to investigate the association between congenital Zika infection and microcephaly [23]. The Brazilian Ministry of Health, the State Health Secretariat of Paraíba, and the U.S. Centers for Disease Control and Prevention (CDC) collaborated on all aspects of the investigation. 

Investigators recruited 592 children for participation in the case-control study from the national microcephaly surveillance system, Registro de Eventos de Saude Publica (RESP)—Microcefalia, from which cases were selected, and the Sistema de Informação de Nascidos Vivos (SINASC), which records all births in Brazil, and from which controls were selected. Investigators classified infants reported to RESP into four mutually exclusive groups according to HC and length at birth: microcephaly (HC ≤ 3rd percentile, HC: length ratio ≤ 0.65), small (HC ≤ 3rd percentile, HC: length ratio > 0.65), disproportionate (HC >3rd percentile, HC: length ratio ≤ 0.65), and no microcephaly (HC >3rd percentile, HC: length ratio > 0.65). Infants were sampled as cases for the case-control study from each of the four RESP groups [23]. Controls were selected to be the same age or younger than the oldest case and matched to cases on geographic location. For the ZODIAC follow-up investigation, eligibility was restricted to children living in macroregions 1 and 2 of Paraíba state because of logistical constraints. The mothers of infants in the case group had to have resided in Paraíba state for 80% of their pregnancy [24]. Children included in the earlier case-control study were eligible for the ZODIAC follow-up investigation if they met the following anthropometric or laboratory criteria:

- Laboratory: non-negative test for Zika-specific neutralizing antibodies in an infant sample, and/or
- Anthropometric: met case-control study criteria for assignment to the microcephaly, small, or disproportionate group [23], as defined above.

**Figure 1:**
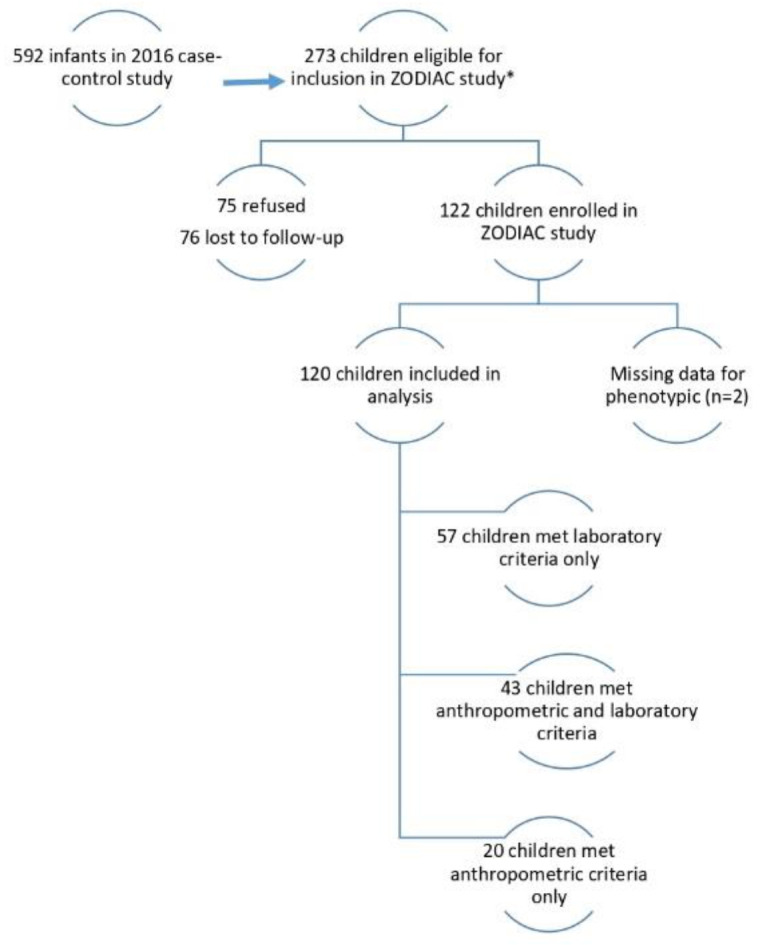
Eligibility for the Zika Outcomes and Development in Infants and Children (ZODIAC) Investigation, Paraíba, Brazil, 2017. Anthropometric criteria: head circumference (HC) ≤3rd percentile for gestational age and sex or HC > 3rd percentile for gestational age and sex and HC-to-body length ratio ≤ 0.65; Laboratory criteria: non-negative test results for Zika-specific neutralizing antibodies in an infant sample.

Figure 1 describes the children included in our analysis. Of the 592 children in the case-control study, 273 children were eligible for the ZODIAC investigation. Of these eligible children, 151 were unavailable for follow-up (75 refused, 76 lost to follow-up), and two were missing phenotype data (see phenotype classifications below), which left 120 children for our analysis. Of the 120 children, 20 met anthropometric eligibility criteria only, 43 met anthropometric and laboratory criteria, and 57 met laboratory criteria only. 

As a surveillance activity, the ZODIAC project was determined not to be research in accordance with the federal human subject protection regulations at 45 Code of Federal Regulations 46.101c and 46.102d, and CDC’s Guidelines for Defining Public Health Research and Public Health Non-Research. The ZODIAC protocol (2.156.321) was approved by the Brazilian National Ethics Committee.

### 2.2. Phenotype Classifications

Diagnosis of Zika virus infection is difficult due in part to the high proportion of asymptomatic infections [25] and the limitations of the laboratory testing [26]. All children participating in the ZODIAC investigation had some suggestive evidence of congenital Zika infection. A subset had the craniofacial phenotype that represents the most severe manifestation of this infection and has been termed “congenital Zika syndrome (CZS)” [7]. Children were categorized by craniofacial phenotype because reduced HC is a syndrome feature [7] and because of the possibility that HC growth in children without this distinctive phenotype might vary according to infection status. A clinical geneticist (C.A.M.) who had identified infants with Zika virus-associated dysmorphology for the original case-control study re-reviewed photographs of the children to confirm typical Zika phenotypes [8] or nonspecific dysmorphic features. For the purpose of this study, children were classified into the Zika-specific phenotype if they had the distinct dysmorphic features of CZS; a nonspecific dysmorphic phenotype if they had dysmorphic features, some of which might be associated with congenital Zika infection (e.g., sloping forehead, frontal narrowing, disproportionately small head or small overall for age and sex, deeply set eyes, and esotropia); and a nondysmorphic phenotype if they had no dysmorphic features.

### 2.3. Ages and Stages Questionnaire

The Ages and Stages Questionnaire—3rd Edition (ASQ-3) is used to screen young children for developmental delays [27]. The ASQ-3 is a caregiver-report instrument that aids the clinician in determining if a child’s development is on schedule, identifies children at risk for developmental delay, and encourages caregiver involvement in supporting the child’s development. The ASQ-3 has five domains: communication, gross motor, fine motor, problem solving, and personal-social. ASQ-3 questionnaires were translated into Brazilian Portuguese and validated in Brazil [28]. We used an amended protocol to administer ASQ-3 questionnaires that were appropriate to a child’s developmental attainment, instead of beginning the assessment with the questionnaire designed for a child of the same chronological age, to account for severely affected children. The amended protocol involved starting with the 6-month interval, regardless of age, and depending on the child’s functional skills, moving back or moving up by one age interval at a time, until an age interval questionnaire was administered for which the child has the abilities assessed by some but not all questions in a domain [29]. We calculated the ASQ *z*-score on each domain for every child using the child’s domain score to compare to the mean and standard deviation of Brazilian children who received that same questionnaire [28].

### 2.4. Head Circumference, Length, and Weight Assessment

Newborn measurements of HC (cm), length (cm), and weight (kg) assessed using a Brazilian Ministry of Health protocol were obtained from registry and medical records. We used data collected for the previous case-control study, including birth HC and length information obtained from medical and RESP (microcephaly registry) records, and results of clinical and laboratory evaluation at 1−7 months of age [23]. In addition, we measured HC, length, and weight when conducting the comprehensive health and developmental evaluations of children for the ZODIAC investigation follow-up at 19–26 months of age.

For the ZODIAC investigation, licensed physicians performed growth, ophthalmologic, and physical exams. Children with vision and hearing impairment and/or neurologic findings were referred for further evaluation. Information collected from the current medical records included HC, length, and weight. The *z*-scores for anthropometric measures at birth were calculated using the INTERGROWTH-21st standards [30], and *z*-scores for subsequent measurements were computed according to World Health Organization (WHO) standards [31].

### 2.5. Analysis Methods

Our objective was to identify infant growth parameters predictive of developmental delay for each of the five ASQ-3 domains. To achieve our objective, we developed models for the five developmental ASQ-3 domain outcomes, quantified as developmental quotient (DQ) *z*-scores, as a function of phenotype features for HC and length. The developmental delay models examined each of the five ASQ-3 domains as a function of HC and length, in addition to HC and length rates of growth and other potential predictor variables. Missing HC and length measurements, and instantaneous rates of HC and length growth were estimated using separate models for HC and length, and used as predictor variables in the ASQ-3 domain specific models (details in Supplemental Material). 

### 2.6. Developmental Quotient *z*-score

Our outcome was developmental delay, as quantified by the DQ *z*-score for each ASQ-3 domain (communication, gross motor, fine motor, problem solving, and personal-social). The ASQ-3 domain scores were converted to DQ *z*-scores using the method described by Attell et al. [29] and Kotzky et al. [32]. For each of the 5 domains, we categorized three developmental delay groups using DQ *z*-scores as defined in the United States [33]. Delay groups were defined as: no delay (greater than −1 standard deviation, SD), possible delay (−1 to −2 SD), and likely delay (less than −2 SD) [27]. We categorized developmental delay across all domains by classifying children into global delay groups: none (<1 SD below mean on all five ASQ-3 domains), severe (≥2 SD below mean on ≥2 domains) and mild to moderate (the remaining children, i.e., ≥2 SD below mean on one domain, or 1–1.9 SD below mean on one to five domains).

### 2.7. Predictor Variables

Predictor variables considered in our HC, length, and developmental delay models are described in detail in the Supplemental Material. To account for missing HC and/or length measurements at birth for some children, the final developmental delay models for DQ *z*-score included the predicted HC and length at birth (cm), and HC and length rate of growth at birth (cm/month). 

### 2.8. Developmental Quotient *z*-score Modeling Using Head Circumference and Length

Modeling each DQ *z*-score domain as a function of HC and length was a two-step process. First, we modeled the repeated HC and length measurements using a Bayesian bivariate normal random-effects (BVN) model (see Supplemental Material). For HC and length for all children included in our BVN model, birth was time zero. There have been many growth models developed and we determined the basic model structure developed by Karlberg [34] for infants adequately described our data. We also considered the first and second order growth models developed by Berkey and Reed [35] and used recently by Surén et al [36]. We used the BVN model to account for the correlation (dependency) between HC and length, to predict birth HC and length, and instantaneous rates of growth of HC and length at birth for each child. We reduced our model by removing variables with low predictive ability, defined as the 90% credible interval (CI) for the parameter estimate including zero, until we had parsimonious models. Second, we modeled the DQ *z*-score for each domain as a function of the predicted birth HC and length (size in cm, and rate of growth in cm per month) simultaneously with the BVN HC and length model. 

The 120 children each had three to four available HC and body length measurements from birth to the 24−26 month follow-up (for a total of 450 measurements), and each child’s DQ *z*-score was measured once at approximately age 24 months (120 measurements for each ASQ-3 domain). Our data included missing values for HC (38) and length (12) and missing data for mother smoking during pregnancy (8), previous children (16), and weight-to-length ratio at birth (37). All missing data were treated as additional parameters to be estimated within our Bayesian models. We had no prior knowledge of the expected parameter values so we used diffused priors for all parameters (N(0, var = 1e6)) and variance components (igamma (0.01, 0.01)). We used 10,000 samples for burn-in, and 200,000 samples post burn-in, with thinning set to four, for a total sample size of 50,000 to summarize the posterior distributions. We examined model fit using plots of observed data versus model predictions. All analyses were conducted using SAS™ software version 9.4.

## 3. Results

Demographic characteristics of the 120 children who participated in the ZODIAC investigation reveal equivalent numbers of females and males, predominantly with weight-to-length ratios < 16, and few premature births (11.7%) (Table 1). Most children (62.5%) had a nondysmorphic phenotype, and there were 19.2% in the Zika-specific and 18.3% in the nonspecific dysmorphic groups. Primary caregivers usually had less than high school education, few smoked during pregnancy, many had a previous child (60.0%), and most breastfed their child for more than 12 months (>80%). Twenty-one of the 23 children classified in the Zika-specific phenotype group had birth HC measurements <3rd percentile for their age and sex and two had postnatal-onset microcephaly. These 23 children had microcephaly at the post-birth follow-up assessment. There are 22 children who were classified as having a nonspecific dysmorphic phenotype, which includes two with microcephaly at birth and one with postnatal-onset microcephaly. Seventy-five children were classified as having a nondysmorphic phenotype, 59 (78.7%) did not have microcephaly at any assessment; seven were classified as having microcephaly at birth but had normal HC at two follow-up assessments. Fourteen of the children were missing HC at birth (five nonspecific dysmorphic, nine nondysmorphic), but had normal HC measurements at approximately age 24 months.

**Figure 2:**
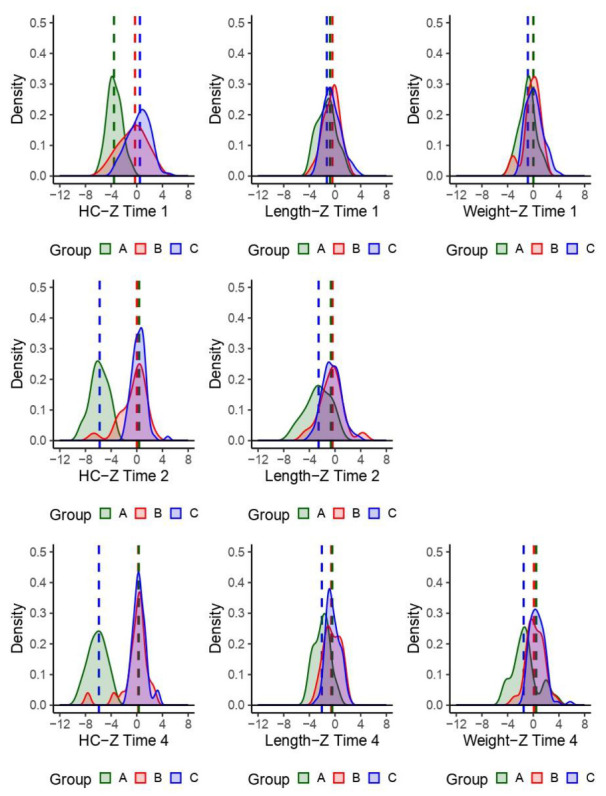
Density plots of observed children’s head circumferences (HC), length, and weight *z*-scores. Time 1: birth; time 2: age 1−7 months; time 4: age approximately 24 months. Group phenotype: A = Zika-specific, B = nonspecific dysmorphic, and C = nondysmorphic.

We evaluated HC, length, and weight over time, by phenotype and child (Figure 2). The HC medians for children with nonspecific dysmorphic and nondysmorphic phenotypes were similar and the median HC was substantially less for children with the Zika-specific phenotype. The HC overlap among the Zika-specific and nondysmorphic groups decreased over time. Weight measurements were not part of the case-control study so there are only two available weight time point measurements. The distributions and median birth weight for the phenotype groups were similar at birth, with the Zika-specific phenotype slightly lower at approximately 24 months of age. 

**Figure 3:**
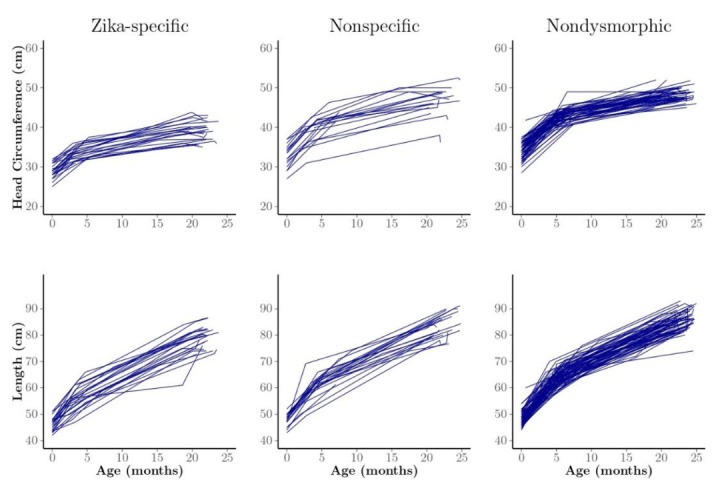
Profile plots of head circumference (HC) and length of observed individual children, generally measured three times at approximately birth, 1−7 months of age, and 24 months by phenotype group. We classified the children into three phenotype groups: Zika-specific, nonspecific dysmorphic, and nondysmorphic.

Profile plots of HC by child and phenotype illustrate greater HC variability in the nonspecific dysmorphic phenotype group, with few children having trajectories like those of the Zika-specific phenotype group (Figure 3). We observed little difference in length among and within the phenotype groups over time (Figure 3).

We assessed the phenotype and DQ *z*-scores by ASQ-3 domain at age 19−26 months (Table 2). Children in the nonspecific dysmorphic phenotype group more frequently had a likely or possible delay on any domain compared to those with a nondysmorphic phenotype. The delay differences for these two groups was largest in the personal-social, problem solving, and gross motor domains. All children with the Zika-specific phenotype had severe global developmental delay, whereas approximately 9% of children in the other two groups had severe global developmental delay. Among the nonspecific dysmorphic and nondysmorphic phenotype groups, 63.6% and 37.3% of children had mild-to-moderate global developmental delay, respectively. 

**Figure 4:**
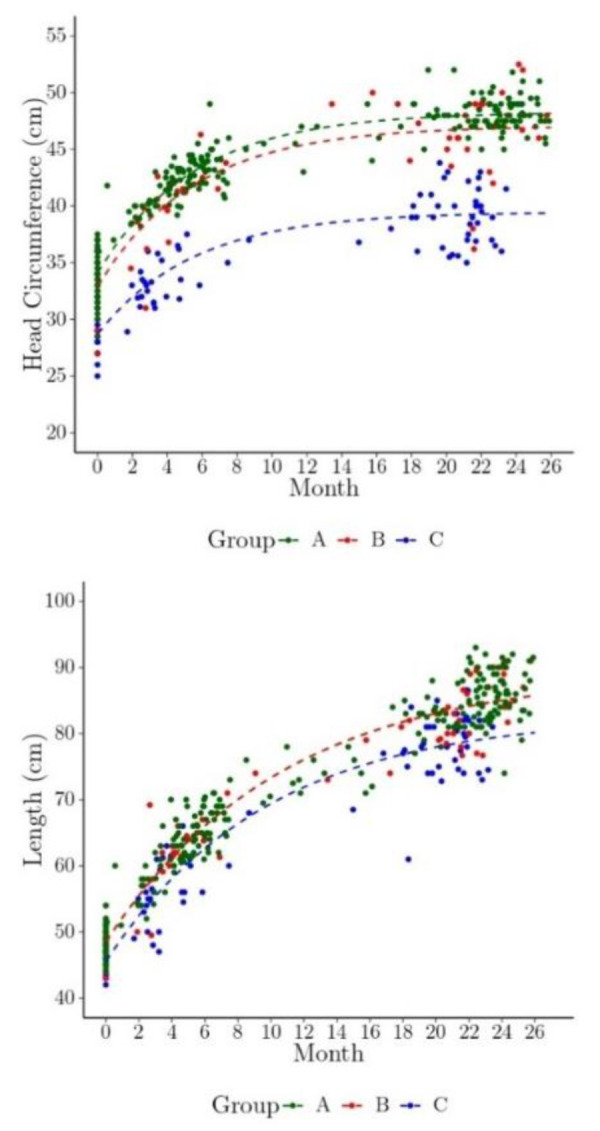
The model predicted marginal head circumference (HC) and length over time (shown here as dashed lines overlaid on the individual children’s observed HC and length measurements, by phenotype group). Children were usually measured three times approximately at birth, 1–7 months of age, and 24 months. Group phenotype: A = Zika-specific, B = nonspecific dysmorphic, and C = nondysmorphic.

Figure 4 shows the predicted means of HC and length by Zika phenotype group overlaid on the observed data for the HC and length models. Note that these predictions account for the dependency of the HC and length within a child by estimating the correlation and using it in the model predictions. The predicted HC plot illustrates the separation between the Zika-specific phenotype group and the nonspecific dysmorphic and nondysmorphic groups, and this separation is less pronounced for length. Children with a nonspecific dysmorphic phenotype exhibit smaller HC relative to those in the nondysmorphic group, but no substantial difference is evident in length among these groups. 

**Figure 5:**
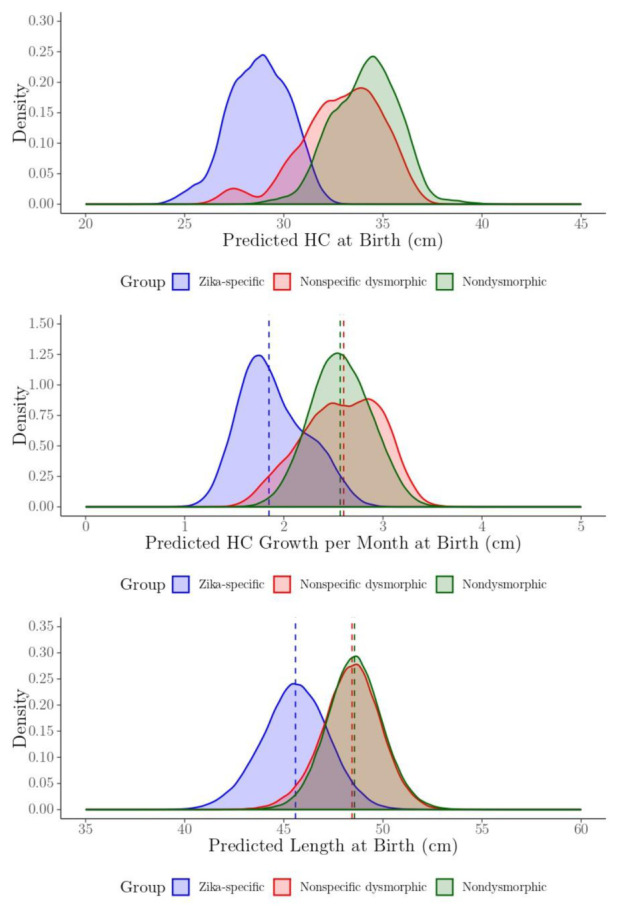
The posterior density plots of the predicted head circumference (HC) and length at birth (cm), and HC instantaneous rate of growth at birth (cm/month) for each child by phenotype group.

Posterior density plots for the predicted HC and length at birth, and predicted HC growth rate per month at birth for each child by phenotype group, are presented in Figure 5. Results illustrate the posterior density plots for the Zika-specific phenotype group are shifted downward for the HC and length at birth, and the HC growth per month at birth is smaller compared to the nonspecific dysmorphic and nondysmorphic groups. 

Modeling results indicate that HC at birth, HC instantaneous rate of growth at birth, and length at birth are predictive of developmental delay (Table 3). The HC at birth and associated rate of growth were the strongest predictors of developmental delay for all ASQ-3 domains. Length at birth and length growth rate were predictive but less consistent than HC across the ASQ-3 domains. We estimated the DQ *z*-score for each ASQ-3 domain at one, two, and three SD below the mean. For example, under the communication domain, the model estimated that a child with a HC 2 SD below the mean (–2SD HC = 27.45 cm, mean = 32.87cm) would have a DQ *z*-score of approximately −4. Birth HC had the largest effect on the DQ *z*-score for each of the five domains, followed by HC instantaneous rate of growth, and birth length, respectively. The estimated effect per one SD decrease from the mean HC were reduced DQ *z*-scores on the communication (−1.30), gross motor (−1.57), fine motor (−1.67), problem solving (−1.29), and personal-social (−1.59) ASQ-3 domains. For each one SD decrease from the mean in HC rate of growth, there were reduced DQ *z*-scores ranging from −1.27 SD (gross motor) to −0.86 SD (communication). Length had the smallest effect, ranging from −0.46 SD (problem solving) to −0.17 SD (gross motor) per one SD decrease below the mean. 

## 4. Discussion

Our results illustrate that early growth parameters are predictive of developmental delay among children conceived during a Zika virus outbreak and likely exposed to Zika virus in utero, who met anthropometric and/or laboratory criteria for Zika virus infection [24]. Identifying whether children without the Zika-specific phenotype were congenitally infected with Zika virus was challenging, and prevented the specific attribution of observed early growth patterns to Zika virus infection. No nucleic acid testing was performed on postnatal samples, and infants were residing in areas where there was Zika virus circulation when serologic testing was performed at 1−7 months of age. Therefore, when laboratory findings suggested infection, we assumed, but were unable to confirm, that infection occurred prenatally. Our results suggest that among children for whom uncertainty about Zika infection status is likely to be greatest, changes in growth parameters over time may be useful in distinguishing between those at risk and not at risk of developmental delay.

Brain imaging has been explored for its potential to identify children with congenital Zika infection who do not have microcephaly and to predict developmental delay. Aragao et al. [37] used brain computed tomography (CT) and magnetic resonance imaging (MRI) scans of infants 1 year of age or younger, to find infants with less extreme brain damage and to compare them with infants with microcephaly. The infants in this study were possible (suspected), probable, or confirmed CZS cases. In three toddlers for whom other infectious causes were ruled out and who were not born with and did not develop microcephaly postnatally, they found asymmetric polymicrogyria, mainly in the frontal lobes, calcifications restricted to the cortical/white matter junctions, mild ventricular enlargement, and delayed myelination. These children had nonspecific clinical signs of brain impairment in infancy that might otherwise have been missed. However, it is important to note that not all of these infants had laboratory tests that suggested they were congenitally infected. Lopes Moreira and colleagues [18] found a statistically significant association between abnormal postnatal brain imaging findings and developmental delay, as assessed by the Bayley Scales of Infant and Toddler Development, third edition (Bayley-III), in toddlers exposed to Zika virus in utero. However, the investigators point out, based on their findings from the same cohort, that non-structural or non-specific findings from brain imaging in neonates with congenital Zika virus exposure are not necessarily predictive of developmental delay [18,19].

It is unlikely that neuroimaging alone will be useful in identifying children for more intensive developmental monitoring in future Zika outbreaks because neuroimaging is not available in many settings, including in northeastern Brazil during the 2015−2016 outbreak, and may not be indicated if available. In the ZODIAC investigation, only 11 of the 23 children in the Zika-specific phenotype group had CT imaging results available in medical records. Only two children in the nonspecific dysmorphic group and only one in the nondysmorphic group had CT results included in their medical record. A description of the evaluation of all U.S. infants with congenital Zika virus exposure reported to the U.S. Zika Pregnancy Registry in 2016 revealed that only 25% of eligible infants received recommended postnatal brain imaging, potentially missing opportunities for early identification of subclinical neurological abnormalities [38]. Finally, even if neuroimaging is available and indicated, the findings may not be specific for congenital Zika infection nor a strong predictor of developmental delay [18]. 

A strength of the ZODIAC investigation is that children had been selected for an earlier case-control study from population-based registries [23] and they, therefore, had a range of phenotypes, unlike earlier studies that have primarily reported on children referred to centers for neurodevelopmental disorders, a highly selected group. Another strength is that follow-up data were collected at multiple timepoints. We expected that children with the Zika-specific phenotype, whose smaller size and lighter weight in the first year of life have been documented [14,39,40], would remain smaller and lighter over time compared to their peers. These children did remain smaller, but their weight was similar to that of the other children. Plausible explanations for the greater than expected weight in this group include inactivity due to motor impairment, differences in food provided given swallowing difficulties, and feeding as a source of comfort for the family and child. The case series included in this analysis may not represent all children who met the eligibility criteria for follow-up, given that 151 of the eligible children did not participate (parents refused or the children were lost to follow-up). Our investigation was also limited by an inability to obtain complete medical histories from available records, and to accurately identify infected children as well as rule out other infectious or noninfectious causes of developmental delays. Nonetheless our results may be useful because children at risk of developmental delay will benefit from receipt of early intervention services, regardless of whether the causal mechanism can be definitively established. Our findings that HC and HC growth rate are good predictors of developmental delay suggest that accurate and frequent measurement of HC may be useful as an indicator for early referral for neurologic evaluation among children conceived during future outbreaks. Current recommendations for the evaluation of infants with possible congenital Zika virus infection include monitoring HC along with other growth parameters as part of a comprehensive physical examination at birth and at each well-child visit [17]. HC growth rate is typically largest in the first six months of life; thus, these early months may be an optimal period for HC monitoring.

Even if the accuracy of diagnostic tests improves, it is likely that congenital Zika virus infection will remain as difficult to diagnose in future outbreaks as in the 2015−2016 outbreak in northeastern Brazil because of the high proportion of asymptomatic infections. Monitoring of early growth parameters may be useful in identifying risk for developmental delay, whether due to an infectious etiology, such as Zika virus infection, a neurodegenerative condition such as Rett syndrome, an anatomic condition such as craniosynostosis, or conditions caused by another etiology associated with decreased or altered brain growth. These findings underscore American Academy of Pediatrics recommendations (https://brightfutures.aap.org/materials.and-tools/guidelines-and-pocket-guide/Pages/default.aspx) that routine monitoring of head circumference and other growth parameters should occur in routine well visits, to ensure that children are identified as early as possible.

## Figures and Tables

**Table 1:** Demographic characteristics for the 120 children and their caregivers in the 2017 Zika Outcomes and Development in Infants and Children (ZODIAC) investigation.

Child Characteristics	Level	N	Percent
Sex	Male	60	50.0
	Female	60	50.0
Zika Phenotype	Zika-specific	23	19.2
	Nonspecific dysmorphic	22	18.3
	Nondysmorphic	75	62.5
Weight-to-length ratio (birth)	<12	17	14.2
	12−16	84	70.0
	>16	6	5.0
	Missing	13	10.8
Premature Child (<37 weeks)	Yes	14	11.7
	No	106	88.3
**Caregiver Characteristics**			
Age (years)	≤ 18	8	6.7
	19−23	29	24.2
	24−28	23	19.2
	29−33	34	28.3
	> 33	26	21.7
Education (years)	≤ 6	24	20.0
	7−8	24	20.0
	9−11	36	30.0
	≥ 12	36	30.0
Smoked during pregnancy	Yes	7	5.8
	No	111	92.5
	Missing	2	1.7
Previous Children	Yes	72	60.0
	No	44	36.7
	Missing	4	3.3
Breastfed Child (months)	0−6	12	10.0
	7−12	8	6.7
	13−18	36	30.0
	19−26	62	51.7
	Missing	2	1.7

**Table 2:** Developmental delay by phenotype group among children with anthropometric and/or laboratory evidence suggestive of possible congenital zika infection, from birth to approximately age 24 months, Zika Outcomes and Development in Infants and Children (ZODIAC) investigation, 2017.

Developmental Classification	Phenotype Group		
	Zika-Specific N (%)	Nonspecific Dysmorphic N (%)	Nondysmorphic N (%)
*Communication*			
<1 SD below mean	0 (0)	14 (63.6)	57 (76.0)
1−1.9 SD below mean	0 (0)	5 (22.7)	11 (14.7)
≥2 SD below mean	23 (100)	3 (13.6)	7 (9.3)
*Gross Motor*			
<1 SD below mean	0 (0)	14 (63.6)	59 (78.7)
1−1.9 SD below mean	0 (0)	5 (22.7)	11 (14.7)
≥2 SD below mean	23 (100)	3 (13.6)	5 (6.7)
*Fine Motor*			
<1 SD below mean	0 (0)	17 (77.3)	63 (84.0)
1−1.9 SD below mean	0 (0)	3 (13.6)	8 (10.7)
≥2 SD below mean	23 (100)	2 (9.1)	4 (5.3)
*Problem-Solving*			
<1 SD below mean	0 (0)	14 (63.6)	61 (81.3)
1−1.9 SD below mean	0 (0)	6 (27.3)	10 (13.3)
≥2 SD below mean	23 (100)	2 (9.1)	4 (5.3)
*Personal-Social*			
<1 SD below mean	0 (0)	15 (68.2)	69 (92.0)
1−1.9 SD below mean	0 (0)	5 (22.7)	2 (2.7)
≥2 SD below mean	23 (100)	2 (9.1)	4 (5.3)
*Development Classification (overall)*			
All domains < 1 SD below mean	0 (0)	6 (27.3)	40 (53.3)
≥2 domains are ≥2 SD below mean	23 (100)	2 (9.1)	7 (9.3)
Remaining	0 (0)	14 (63.6)	28 (37.3)

The overall remaining group is classified as 1 domain ≥2 SD below mean or 1−5 domains, 1−1.9 SD below mean. SD = standard deviation.

**Table 3:** The estimated impact on developmental delay by ASQ-3 domain using the Bayesian head circumference (HC), length, and developmental delay models (expected *z*-score at select growth parameter values)^*^. Centimeter and centimeter per month estimates were averaged across developmental domains.

Growth Parameter	Communication Mean (PI)	Gross Motor Mean (PI)	Fine Motor Mean (PI)	Problem-Solving Mean (PI)	Personal-Social Mean (PI)
*Head Circumference*					
Mean = 32.87 cm	−1.49 (−1.88 to −1.10)	−1.25 ( −1.67 to −0.83)	−1.10 (−1.51 to −0.70)	−1.52 (−1.90 to −1.13)	−1.07 (−1.50 to −0.65)
−1 SD = 30.16 cm	−2.79 (−3.56 to −2.00)	−2.82 (−3.66 to −1.97)	−2.77 (−3.56 to −1.97)	−2.81 (−3.57 to −2.02)	−2.66 (−3.54 to −1.77)
−2 SD = 27.45 cm	−4.10 (−5.48 to −2.67)	−4.40 (−5.91 to −2.87)	−4.44 (−5.86 to −3.01)	−4.09 (−5.49 to −2.65)	−4.25 (−5.84 to −2.63)
−3 SD = 24.74 cm	−5.40 (−7.42 to −3.30)	−5.97 ( −8.15 to −3.72)	−6.10 (−8.19 to −3.99)	−5.38 (−7.43 to −3.31)	−5.84 (−8.19 to −3.47)
*Head Circumference Growth*					
Mean = 2.45 cm/month	−1.49 (−1.88 to −1.10)	−1.25 ( −1.67 to −0.83)	−1.10 (−1.51 to −0.70)	−1.52 (−1.90 to −1.13)	−1.07 (−1.50 to −0.65)
−1 SD = 2.02 cm/month	−2.35 (−3.02 to −1.67)	−2.52 (−3.24 to −1.79)	−2.27 (−2.97 to −1.57)	−2.58 (−3.26 to −1.89)	−2.14 (−2.90 to −1.36)
−2 SD = 1.60 cm/month	−3.21 (−4.37 to −2.04)	−3.80 (−5.06 to −2.50)	−3.44 (−4.65 to −2.23)	−3.63 (−4.81 to −2.42)	−3.20 (−4.55 to −1.83)
−3 SD = 1.17 cm/month	−4.07 (−5.77 to −2.35)	−5.07 (−6.89 to −3.20)	−4.62 (−6.36 to −2.85)	−4.69 (−6.43 to −2.94)	−4.26 (−6.21 to −2.28)
*Length*					
Mean = 47.96 cm	−1.49 (−1.88 to −1.10)	−1.25 ( −1.67 to −0.83)	−1.10 (−1.51 to −0.70)	−1.52 (−1.90 to −1.13)	−1.07 (−1.50 to −0.65)
−1 SD = 46.09 cm	−1.86 (−2.80 to −0.95)	−1.42 (−2.45 to −0.43)	−1.34 (−2.28 to −0.43)	−1.98 (−2.92 to −1.06)	−1.45 (−2.53 to −0.40)
−2 SD = 44.22 cm	−2.24 (−4.00 to −0.53)	−1.60 (−3.55 to 0.25)	−1.58 (−3.34 to 0.11)	−2.44 (−4.23 to −0.71)	−1.83 (−3.88 to 0.14)
−3 SD = 42.35 cm	−2.62 (−5.19 to −0.11)	−1.77 (−4.64 to 0.96)	−1.82 (−4.40 to 0.68)	−2.89 (−5.51 to −0.38)	−2.20 (−5.21 to 0.69)

Expected delay *z*-score when all parameters are equal to their mean values; Abbreviations: SD = standard deviation, PI = 95.0% prediction interval).

## Supplementary Materials

The following are available online at https://www.mdpi.com/2414-6366/5/4/155/s1.
