# Supplementary material for: Early Growth Parameters as Predictors of Developmental Delay among Children Conceived During the 2015–2016 Zika Virus Outbreak in Northeastern Brazil
Source: Trop Med Infect Dis. 2020 Oct 1;5(4):155. doi: 10.3390/tropicalmed5040155 (PMC7709658; doi:10.3390/tropicalmed5040155)
Supplement: Supplementary file 1 [file tropicalmed-05-00155-s001.zip › tropicalmed-915897-supplementary.pdf]

Supplemental Material for Early Growth Parameters as Predictors of Developmental Delay Among Children Conceived During the 2015–2016 Zika Virus Outbreak in Northeastern Brazil: Modeling Developmental Delay

Modeling Overview

Our primary goal is to model developmental delay (DD) as defined by the developmental quotient (DQ) z-scores for the Ages & Stages Questionaire 3rd edition (ASQ-3) communication, gross motor, fine, personal / social, and problem solving as a function of hypothesized predictor variables using a Bayesian approach. We use the method of Attell et al. [29] to estimate the developmental quotient (DQ) z-score. We hypothesized that head circumference (HC) and length, absolute size and rate of growth, would be indicators of future DD. For modeling DD using HC and length growth rate (cm/month) and size (cm) at birth we developed a model to predict the instantaneous rate of growth for length and HC at birth in addition to predicting the birth length and HC. Our model developed for HC is as described by Karlberg et al. [34]. Karlberg proposed an exponential function that could describe the rapid growth in infancy. Here we illustrate the differential equation expression and derive the model described by Karlberg. Our study included data to approximately age 24 months

and therefore this model is adequate for modeling HC and length. However, the differential equation could be modified to allow for a more flexible model to describe growth as a child progresses past the infant stage of life. We illustrate the derivation of the HC and length models below.

Modeling Head Circumference and Length

Let γ represent HC or length, then our model may be expressed as

$$\frac{d\gamma}{dt} = I - \lambda\{\gamma(t) - \gamma(0)\}. \quad (1)$$

Let

$$Y(t) = \gamma(t) - \gamma(0), \quad (2)$$

where I is the intensity, λ is the rate, and $Y(0) = 0$. Then as $\gamma(0)$ is a constant, note $dY/dt = d\gamma/dt$ and the differential equation Y is defined as

$$\frac{dY}{dt} = I - \lambda * Y, \quad (3)$$

$$\frac{dY}{dt} + \lambda * Y = I. \quad (4)$$

Multiplying both sides by $e^{\lambda t}$ and applying the product rule we obtain

$$\frac{d}{dt}[e^{\lambda t}Y] = Ie^{\lambda t}. \quad (5)$$

Integrating both sides with respect to t and placing the constant of integration (c) on the right side results in

$$e^{\lambda t}Y = \frac{I}{\lambda}e^{\lambda t} + c, \quad (6)$$

$$Y = \frac{I}{\lambda} + ce^{-\lambda t}. \quad (7)$$

Note that by definition when $Y(0) = 0$ implies that $c = -\frac{I}{\lambda}$ and leads to

$$Y = \frac{I}{\lambda}(1 - e^{-\lambda t}), \tag{8}$$

which, using equation (2), results in Karlberg's function

$$\gamma(t) = \gamma(0) + \frac{I}{\lambda}(1 - e^{-\lambda t}). \tag{9}$$

Head Circumference and Length Model Properties

There are three properties of our HC and length models (9) that are of interest to explore as predictors for the DD outcomes. First, at $t = 0$, the estimate of HC and length is given by $\gamma(0)$. Second, the estimated HC and length when $t \to \infty$, which is an estimate of the child's final HC and length, is given by

$$\lim_{t\to\infty} \gamma(t) = \gamma(0) + \frac{I}{\lambda}. \tag{10}$$

Third, we are interested in the instantaneous rate of HC and length growth, which is given by equation (1) as

$$\frac{d\gamma}{dt} = I - \lambda\{\gamma(t) - \gamma(0)\},$$

substituting in equation (9) results in

$$\frac{d\gamma}{dt} = I - \lambda\{\gamma(0) + \frac{I}{\lambda}(1 - e^{-\lambda t}) - \gamma(0)\}. \tag{11}$$

Equation (11) can be reduced to

$$\frac{d\gamma}{dt} = Ie^{-\lambda t}, \tag{11b}$$

or expressed as

$$\frac{d\gamma(t)}{dt} = Ie^{-\lambda t}. \tag{11c}$$

We may obtain the instantaneous rate of growth at any desired time by letting $t = c$, where c is the desired time to estimate the growth. Here we set $t = 0$ and and this results in I as the estimated instantaneous rate of growth at birth, which is used as a predictor variable in our DD models.

Head Circumference and Length Random Effects Models

We model HC and length using equation (9) and incorporate random effects for the repeated measurements (t) within individuals. Define $\gamma(0)$, I, and λ as η_0, η_1, and η_2, respectively. Our random effects model is defined as

$$\gamma(t) = \eta_0 + b_0 + \frac{\eta_1 + b_1}{\eta_2 + b_2}(1 - e^{-(\eta_2+b_2)t}) + e, \tag{12}$$

where η_0, η_1, η_2 are defined in the HC and length models as B and A, respectively, and are a function of covariates. Note b_0, b_1, and b_2 are subject-specific random effects, and e is the error term, and all are assumed $N(0, \sigma_{bl})$ and uncorrelated with each other, and $l = 0$, 1, 2, and e. The length model random effects are described below in terms of a_0, a_1, and a_2 instead of b_0, b_1, and b_2, respectively.

Head Circumference (HC) and Length Modeling Procedure

We first analyzed separate models for HC and length over time as a function of the following covariates.

1. Phenotype group (Zika-specific (Grp_1), nonspecific dysmorphic (Grp_2), nondysmorphic)
2. Child's sex (male, female); $Sex = 1$ if female and 0 if male
3. Maternal smoking during pregnancy (yes, no); $Smoke = 1$ if yes and 0 if no
4. Mother given birth to previous children (yes, no); $Child = 1$ if yes and 0 if no
5. Prematurity of the child (yes, no); $Gest = 1$ if yes and 0 if no
6. Amount of time that the mother spent breastfeeding child (measured in months)
7. Weight (kg) to length (cm) ratio (WL) of the child at birth (continuous measurement, not included in length model)

A full model with all covariates using equation (12) was estimated for HC and length over time. A stepwise procedure was used to retain the more predictive covariates and build a parsimonious model. If the estimated posterior 80% credible interval (CI_B) captured zero then the variable was dropped from the model. We added in variables that had been previously dropped after removing subsequent variables and continued this process for the HC and length models until we achieved parsimonious models. This method for reduction was repeated until all variables had a posterior 80% CI_B that did not include zero. The final HC model included the following variables in equation (12) in terms of $\eta_0(B_0)$, $\eta_1(B_1)$, and $\eta_2(B_2)$.

$$B_0 = \beta_{00} + \beta_{01}Grp_1 + \beta_{02}Grp_2 + \beta_{03}Sex + \beta_{04}WL + \beta_{05}Smk + \beta_{06}Child + b_0 \tag{13}$$

$$B_1 = \beta_{10} + \beta_{11}Grp_1 + \beta_{12}Sex + \beta_{13}WL + \beta_{14}Child + b_1 \tag{14}$$

$$B_2 = \beta_{20} \tag{15}$$

where all variables are as defined previously. Our final length model includes the following variables in equation (12) in terms of $\eta_0(A_0)$, $\eta_1(A_1)$, and $\eta_2(A_2)$.

$$A_0 = \alpha_{00} + \alpha_{01}Grp_1 + \alpha_{02}Gest + a_0 \tag{16}$$

$$A_1 = \alpha_{10} + a_1 \tag{17}$$

$$A_2 = \alpha_{20} + \alpha_{21}Grp_1 \tag{18}$$

We fit the final reduced HC and length models as a bivariate normal model using the above variables to account for the intra-child correlation of HC and length. This bivariate model was used to generate model-predicted values of HC at birth (cm), length at birth (cm), instantaneous rate of HC growth at birth (cm/month), and instantaneous rate of length growth at birth (cm/month). These model generated estimates were used as predictor variables in our DD z-score by ASQ-3 domain models.

Developmental Delay Model

Our goal is to model developmental delay (DD) using the method of Attell et al. [29] to estimate the developmental quotient (DQ) z-score. We estimate the DQ z-scores given the ASQ z-scores by 1) estimating the DQ score given the ASQ z-score, 2) adjusting the DQ score based on the child's age and test appropriate age, and 3) converting the adjusted DQ score to a DQ z-score. Our DD model for the jth DQ z-score (j = communication, gross motor, fine, personal / social, and problem solving) as a function of p predictor variables using

$$\phi_{ij} = \beta_0 + \beta_{pi} X_{pij} + e_{ij}, \tag{19}$$

where ϕ_{ij} is the ith child and j^{th} DQ z-score, e_{ij} is assumed $N(0, \sigma_j^2)$, and p = 1, 2, 3 ....

Developmental Delay (DD) Modeling Procedure

We used the following procedure to model DD z-score by ASQ-3 domain using a Bayesian linear model. We generated model predictions for HC and length at birth (equations 15 and 18, respectively), and instantaneous rate of growth at birth for HC and length (equations 16 and 19, respectively). These predictions were used as predictor variables in each of our DD z-score by ASQ-3 domain models, equation (19), by standardizing the predictions using the following method.

$$\tau_{ik} = \frac{\mu_{ik} - \bar{\mu}_k}{\sigma_{\mu(k)}} \tag{20}$$

where k = B_0, B_1, A_0, and A_1 and i^{th} child. The HC and length at birth (cm) are estimated for each child by B_0 and A_0, respectively. The instantaneous rate of growth at birth (cm/month) for HC and length are estimated for each child by B_1 and A_1, respectively. The τ_{ik} quantities are estimated during the Bayesian process for each sample and using these samples the mean, $\bar{\mu}_k$, and standard deviation, $\sigma_{\mu(k)}$, are estimated using the 120 children and resulting standardized estimates, τ_{ik}, are used as the predictor variables in the DD z-score by ASQ domain models. In addition, we considered the following covariates in the

DD z-score models: child blood lead levels, hematocrit, free T4, caregiver's age, caregiver's education, and an indicator for reported maternal Zika symptoms during pregnancy. We performed diagnostics on these models and evaluated model fit and none of these covariates were retained in our final DD z-score models using the previously defined criteria for retaining variables. Hence, after model reduction our final DD z-score models retained HC (B_0) and length (A_0) at birth, and HC instantaneous rate of growth at birth (B_1) as predictor variables. We then summarized the five DD z-score domain models for interpretation.

Missing Data

Fifty-two of the 120 children had at least one missing value for the predictor and outcome variables for all considered models. Ten of these 52 children had missing values for more than one predictor and / or outcome variables. There are 38 HC and 12 length missing values corresponding to 34 and 12 children, respectively. The following predictor variables had missing values: weight to length ratio of child at birth (37 time points for 13 children), maternal smoking during pregnancy (eight time points for two children), and mother given birth to previous children (16 time points for 4 children). All missing values were assumed missing at random (MAR).

In our Bayesian analysis all missing values were treated as random variables, i.e., additional parameters to be estimated and summarized through their posterior distributions. The HC and length models had missing values whereas the DD z-score models had no missing values for the outcome or the considered predictor variables. All predictor variables with missing values were modeled using child's sex and prematurity of the child. Missing weight to length ratio values were modeled using a linear model and missing maternal smoking during pregnancy and mother having previous children values were modeled using a logistic regression model. Missing HC and length values were estimated using the HC and length models.

Bayesian MCMC Procedure

We used the following procedure in SAS™ 9.4 PROC MCMC to simultaneously estimate our HC, length, and DD z-score models (by ASQ-3 domain). We used one chain, 10,000 burn-in samples, and 200,000 samples after burn-in with thinning set to four for a final sample size of 50,000 to quantify the posterior distributions. We ran the chain a long time to ensure we sampled throughout the posterior distributions given the complexity of our model. Note the length and HC bivariate model used repeated measures, 120 children with 450 measurements over time. In contrast, our DD z-score models used one measurement per person, at approximately age 24 months, for 120 measurements (one measurement per child). We used diffused priors of $N(0, \sigma^2 = 1e6)$ for all fixed effects and an $inverse-gamma$ $igamma(shape = 0.01, scale = 0.01)$ for all variance components. We conducted a sensitivity analysis by using different priors, e.g., $igamma(shape = 3/10, scale = 10/3)$ and there were no substantial differences in the estimated parameter posterior distributions.
